# Cell biology as the basis of a better understanding of cancer

**DOI:** 10.1186/1475-2867-5-33

**Published:** 2005-11-30

**Authors:** Denys N Wheatley

**Affiliations:** 1 Director of BioMedES, Leggat House, Keithhall, Inverurie, Aberdeen AB51 0LX, UK

## Abstract

Clinicians will argue that cancer can only really receive the treatment that is needed through thorough understanding of medicine. However, even empirical approaches to therapy result in experimental analysis of the agencies involved on test cells, usually in culture. From the obverse perspective, cell biologists will argue that until we fully understand cell cycle regulation, tumour management will be too imprecise to make the best advances. A forum is needed whereby the fundamental studies on cells prior to, during and after transformation in vitro can be freely reported (open access) and discussed. The action of anticancer agents and cancer preventative substances can more easily be studied in vitro before the often excessive complexity of making similar studies in experimental and human cancers is tackled. *Cancer Cell International *is committed to providing such a forum. Ironically within a few months of launching this open access journal, Elsevier had much the same idea, and there one has to pay for the privilege of downloading vital papers in this biomedical field.

Cancer journals have an interesting history. Many of them are the official journals from established institutions or societies that have a fine track record in research and medical practice, with an avid and faithful readership. There are probably more journals devoted to cancer than any other field of medical science. The reason is obvious in that cancer is a scourge, a disease that manifests itself in a vast array of different forms and affects young and old human beings, animals, and plants. Perhaps one of the most remarkable things in the whole of biology is the development of an adult organism from an egg, but during this process or at some stage after it is complete, some cells lose their co-ordinates and start to grow anomalously in a relatively unregulated manner. They will continue to grow in circumstances where normal cells would be constrained. So the problem being addressed is one that comes down to the very heart of cellular biology, to the regulation of the cell cycle, the process of differentiation and the control at the next level of organisation in the development of tissues, organs and bodies. Why is it that cancer cells carry on dividing under circumstances where normal cells become constrained? It is a keen academic problem because we seek to find out from the pathology what has gone wrong.

In many ways, advances in cancer research have been rapid largely because we have begun to understand fundamental cell cycle control, genetic alterations consistent with transformation, and the ways in which drugs interact with tumour as well as normal cells. Controversy continues; some believe it is not so much that a series of lesions occur within individual cells themselves as a case of inappropriate communication that leads to "a society of cells" breaking away from the "normal" constraints when certain stresses are placed upon them. Looked at this way, it is perhaps surprising that cancer is not even more rife than we find, especially among longer lived species.

Whatever way we wish to view cancer, it continues to attract good research funding because, although cancer is not the major killer amongst mankind, it is a frightening (malignant) disorder that has for many years not seen novel and better therapies emerging An exception is seen with cervical cancer, but the cost of regular screening and reading of exfoliative cytology samples is both a time-consuming and expensive business, which was supported from early days and has traditionally been maintained.

Much work can be done not only on human and animals cells that have a malignant phenotype, but on quite remote organisms such as yeast and fungi, which give information about control mechanisms and their derangement. The use of animals to test carcinogens and cancer drugs has been drastically reduced, bringing cell models including spheroids (3-D cultures) much more into vogue. The number of articles being produced on cancer each year around the world has reached quite phenomenal proportions, with many journals being devoted to it. But some of those that in recent times used to "serve" the grass-root work on fundamental cancer research have become increasingly selective, in that average to good studies are seen as not sufficiently high profile for the prestigious pages of the best known journals. The result is that many researchers will inevitably have to seek other journals that do not indulge so heavily in "cherry-picking". We also need to have fast publication with open access to the readers without the need to pay (the Varmos principle).

*Cancer Cell International *was set up with the specific intention of addressing some of the above problems, and was one of the very first niche or specialist – and now referred to as "independent" journals – at BioMed Central . My aim has been to have a journal immediately available to everyone worldwide through the internet that is devoted **to the cellular aspects of cancer**. Some four months after launch, Elsevier had the same idea and launched *Cancer Cell*. Cell Press, now part of Elsevier, had developed a strong track record with its publication of *Cell *and *Neuron*. Our position turned almost immediately into a David and Goliath encounter, and the saving grace of David persisting is that you have the continued opportunity of an online, immediately accessible, free of charge journal, unlike *Cancer Cell*, which is both exclusive and expensive. Compare the costs of getting a paper into *Cancer Cell *– if you are so lucky – with that of getting it into *Cancer Cell International*. [Forget for a moment the impact factor problem – almost all articles wherever published can be accessed one way or another these days, so it's the article that counts, not the journal.] I call upon those of you who do good scientific work in the field of cancer cell biology to submit manuscripts online to us, and have the type of journal that meets modern demands fairly across the board and for countries less advantaged than those privileged ones of North American and Western Europe. It will not exclude the average and good in order to concentrate on the best. It will review all papers *fairly*, wherever they come from.

We have no page limit on the number of papers we can publish per month or year. Colour is not an issue, and you retain the copyright of your article. So, why not make the most of this opportunity. We would greatly appreciate seeing your reports, and if you need presubmission appraisal, we can advise you on this matter before you submit online.

